# Impact of the new pulmonary hypertension definition on long‐term mortality in patients with severe aortic stenosis undergoing valve replacement

**DOI:** 10.1002/clc.23685

**Published:** 2021-07-04

**Authors:** Micha T. Maeder, Lukas Weber, Daniel Weilenmann, Joannis Chronis, Lucas Joerg, Susanne Pohle, Philipp K. Haager, Martin Brutsche, Thomas Neumann, Otto D. Schoch, Hans Rickli

**Affiliations:** ^1^ Cardiology Division Kantonsspital St. Gallen St. Gallen Switzerland; ^2^ Respiratory Medicine Division Kantonsspital St. Gallen St. Gallen Switzerland; ^3^ Rheumatology Division Kantonsspital St. Gallen St. Gallen Switzerland

**Keywords:** aortic valve replacement, cardiac catheterization, pulmonary artery wedge pressure, pulmonary vascular resistance

## Abstract

**Background:**

The new 2018 pulmonary hypertension (PH) definition includes a lower mean pulmonary artery pressure (mPAP) cut‐off (>20 mmHg rather than ≥25 mmHg) and the compulsory requirement of a pulmonary vascular resistance (PVR) ≥3 Wood units (WU) to define precapillary PH. We assessed the clinical impact of the 2018 compared to the 2015 PH definition in aortic stenosis (AS) patients undergoing aortic valve replacement (AVR).

**Methods:**

Severe AS patients (n = 487) undergoing pre‐AVR right heart catheterization were classified according to the 2015 and 2018 definitions. Post‐AVR mortality (median follow‐up 44 months) was assessed.

**Results:**

Based on the 2015 definition, 66 (13%) patients exhibited combined pre and postcapillary PH (CpcPH), 116 (24%) isolated post‐capillary PH (IpcPH), 28 (6%) precapillary PH, and 277 (57%) no PH at all. Overall, 52 (11%) patients were reclassified: 23 no PH into IpcPH; 8 no PH into precapillary PH; 20 precapillary PH into no PH; 1 CpcPH into IpcPH. By the 2015 definition, only CpcPH patients displayed increased mortality, whereas by the 2018 definition, precapillary PH patients also experienced higher mortality than those without PH. Among the PH definition components, PVR ≥3 WU was the strongest predictor of death (hazard ratio > 4).

**Conclusions:**

In severe AS, a higher number of IpcPH patients are diagnosed by the 2018 definition, even though they have the same prognosis as those without PH. Patients with true precapillary PH are more accurately identified by the 2018 definition that includes a pulmonary vascular disease criterion, that is, PVR ≥3 WU, a strong mortality predictor.

## 1 INTRODUCTION

The 2015 European Society of Cardiology (ESC)/European Respiratory Society (ERS) guidelines define any pulmonary hypertension (PH) as a mean pulmonary artery pressure (mPAP) ≥25 mmHg, and a mean pulmonary artery wedge pressure (mPAWP) ≤15 mmHg versus >15 mmHg is applied to differentiate precapillary from post‐capillary PH.^1^ It has, however, been argued that the mPAP cut‐off of 25 mmHg is too high, given that the upper limit of a normal mPAP is approximately 20 mmHg,^2^, ^3^ and because studies have revealed higher mortality in patients with mPAP 21–24 versus ≤20 mmHg,^4^, ^5^ as well as in patients with mPAP 19–24 versus ≤18 mmHg.^6^, ^7^ In addition, the definition of precapillary PH solely based on an elevated mPAP in combination with a nonelevated mPAWP, that is, ≤15 mmHg, was criticized, because this definition does not include a marker of a pulmonary vascular abnormality.^3^ The 6th 2018 PH World Symposium, therefore, proposed a new PH definition including the following key elements: (1) lower mPAP cut‐off (>20 mmHg rather than ≥25 mmHg); (2) compulsory requirement of pulmonary vascular resistance (PVR) ≥3 Wood units (WU) for defining precapillary PH.^3^ In addition, the 2018 definition suggests not using any longer the diastolic pressure gradient (DPG) for differentiating isolated post‐capillary (IpcPH) from combined pre and postcapillary PH (CpcPH), but instead only the PVR ≥3 WU criterion (previously: PVR >3 WU).^8^ The underlying reasons are as follows: (1) high prevalence of negative DPG values that cause confusion; (2) contradictory data concerning the prognostic value of DPG (recently summarized by Lang^9^).^8^

This new definition is under intense discussion.^10^, ^11^, ^12^, ^13^ Arguments against adopting it include potential PH overdiagnosis and overtreatment, and that the change in definition affected only the mPAP cut‐off, whereas the mPAWP and PVR cut‐offs for hemodynamic group classification were left unaltered despite evidence showing that the upper limit of normal may possibly be lower for these parameters.^10^ For example, it has been shown recently that the risk of death is already rising at a PVR cut‐off of 2.2 WU.^14^ There is, however, still little data on the clinical impact of this new PH definition in terms of reclassification rates and prognosis.^15^, ^16^, ^17^ More particularly, the new PH definition's role in patients with aortic stenosis (AS) is unknown. The presence and type of PH according to the 2015 definition have been demonstrated to predict mortality in AS patients undergoing aortic valve replacement (AVR).^18^, ^19^, ^20^, ^21^, ^22^ In this context, however, the new PH definition's role has not yet been investigated. In the current study, we assessed the new PH definition's clinical impact in a large patient population with severe AS undergoing AVR with respect to reclassification rate and prognostic impact.

## 2 METHODS

### 2.1 Study population

This is a retrospective analysis of prospectively and systematically collected cardiac catheterization data.^19^ Overall, 487 consecutive patients with severe AS undergoing cardiac catheterization prior to AVR in a single center between January 2011 and January 2016 were included. Complete data on mPAP, mPAWP, PVR, and DPG were available for all patients so that classification could be performed according to both the 2015^1^ and 2018^2^ definition. The study was approved by the ethics committee of the Canton of St. Gallen (project number 2016–02113). Owing to its retrospective design, a waiver of consent was granted.

### 2.2 Cardiac catheterization

Procedures were generally (>95%) performed between 8 and 10 a.m., with the patient in the fasting state and after withholding loop diuretics and renin‐angiotensin system inhibitors. Patients underwent coronary angiography using 5‐ or 6‐French catheters via femoral or radial artery access, and right heart catheterization using 6‐French Swan‐Ganz catheters via femoral or brachial access. Routine right heart catheterization at the time of coronary angiography has been our practice for more than 20 years in patients with AS evaluated for AVR. The midthoracic level was used as zero reference point. Systolic pulmonary artery pressure (sPAP), diastolic pulmonary artery pressure (dPAP), mPAP, and pulmonary artery wedge pressure were measured. Measurements were obtained at end‐expiration, while avoiding patient's breath‐holding and especially Valsalva maneuvers, with the mean of three to five measurements taken. The mPAWP was calculated over the entire cardiac cycle, and V waves were included. This practice leads to higher values compared to the measurement of the end‐diastolic pulmonary artery wedge pressure. However, for the estimation of the impact of the left heart contribution to pulmonary pressures and calculation of PVR respectively, mPAWP is preferred.^23^ In patients with atrial fibrillation, at least five cardiac cycles were used to average pulmonary artery pressure and pulmonary artery wedge pressure. Cardiac output was assessed by the indirect Fick method based on blood gasses, with blood samples taken in duplicate via arterial access and pulmonary artery catheter. Diastolic pressure gradient was calculated as the difference between dPAP and mPAWP, and transpulmonary gradient as that between mPAP and mPAWP, and PVR as transpulmonary gradient divided by cardiac output. All pressure readings were double‐checked by the operator using manual review of the pressure tracings before recording them into the report.

### 2.3 Hemodynamic definitions

According on the 2015 ESC/ERS guidelines,^1^ PH is defined as mPAP ≥25 mmHg; IpcPH as mPAP ≥25 mmHg, mPAWP >15 mmHg, PVR ≤3 WU and/or DPG <7 mmHg; CpcPH as mPAP ≥25 mmHg, mPAWP >15 mmHg, PVR >3 WU and/or DPG ≥7 mmHg; precapillary PH as mPAP ≥25 mmHg and mPAWP ≤15 mmHg (i.e., without a PVR criterion). Since according to this original ESC/ERS definition, there are unclassifiable patients (i.e., those with discordant PVR and DPG: PVR ≤3WU but DPG ≥7 mmHg or PVR >3WU but DPG <7 mmHg), IpcPH was defined as PVR ≤3 WU and DPG <7 mmHg, and CpcPH was defined as PVR >3 WU and/or DPG ≥7 mmHg. This approach is supported by the recent observation that among patients with PH in the context of left heart disease, those with PVR >3 WU and/or DPG ≥7 mmHg had a similar prognosis as those with PVR >3 WU and DPG ≥7 mmHg, whereas both groups had worse survival than those with PVR ≤3WU and DPG <7 mmHg.^24^ According to the 2018 proposal^3^, ^8^ IpcPH is defined as mPAP >20 mmHg, mPAWP >15 mmHg, and PVR <3 WU; CpcPH as mPAP >20 mmHg, mPAWP >15 mmHg, and PVR ≥3 WU; precapillary PH as mPAP >20 mmHg, mPAWP ≤15 mmHg, and PVR ≥3 WU. Patients with mPAP >20 mmHg, mPAWP ≤15 mmHg, and PVR <3 WU do neither fulfill criteria for precapillary PH nor for post‐capillary PH. This group of patients is not addressed in the 2018 definition papers,^3^, ^8^ and it is not explicitly stated either whether any patient with mPAP >20 mmHg should be classified as having PH. Therefore, we assumed that these patients do not have PH as there is not clear evidence for pulmonary vascular disease and/or elevated left atrial pressure.

### 2.4 Follow‐up

All patients underwent surgical (71%) or transcatheter (29%) AVR following a median interval of 21 (12–35) days post‐catheterization. Information on long‐term follow‐up was obtained from patients, general practitioners, and hospital or practice cardiologists. The endpoint was all‐cause mortality.

### 2.5 Statistical analysis

Categorical data were presented as numbers and percentages, and continuous data as mean ± standard deviation or median (interquartile range), as appropriate. Patients from different hemodynamic PH categories and those without PH according to the 2015 and 2018 definitions were compared using chi‐square tests, analysis of variance, or Kruskal‐Wallis tests, as appropriate. Survival of patients from different PH groups and different hemodynamic categories were compared using Kaplan–Meier plots and log‐rank tests. Cox regression was applied to describe the association between variables of interest and mortality. A *p*‐value <.05 was considered statistically significant. Analyses were performed using SPSS statistical package Version 20.0 (SPSS Inc., Chicago, IL, USA).

## 3 RESULTS

### 3.1 Study population

The mean age of the 487 patients was 74 ± 9 years, and 57% were males. The indexed aortic valve area was 0.42 ± 0.12 cm^2^/m^2^, and the left ventricular ejection fraction 58 ± 12%. The mean mPAP, mPAWP, and PVR were 26 ± 10 mmHg, 16 ± 8 mmHg, and 2.2 ± 1.3 WU, respectively, and the median (interquartile range) DPG was 0 (−3–2) mmHg.

### 3.2 2015 ESC/ERS definition

According to the 2015 definition, there were 210 (43%) patients with PH, 66 (13%) of whom exhibited CpcPH, 116 (24%) IpcPH, and 28 (6%) precapillary PH, whereas 277 (57%) patients had no PH at all. In only one CpcPH patient, the diagnosis was based only on the DPG criterion, while all others displayed a PVR >3 WU. Patient characteristics and hemodynamics of patients with CpcPH, IpcPH, precapillary PH, and those without PH according to the 2015 definition are shown in Table S1.

### 3.3 2018 World symposium definition

The number of patients with PH by the 2018 definition and the difference with the 2015 definition was 221 (45% of all patients) and 11 respectively; 65 (13%; −1) of whom exhibited CpcPH, 140 (29%; +24) IpcPH, and 16 (3%; −12) precapillary PH, whereas 266 (55%; −11) patients had no PH at all. Patient characteristics and hemodynamics of patients with CpcPH, IpcPH, precapillary PH, and those without PH according to the 2018 definition are shown in Table 1.

**TABLE 1:** Clinical characteristics, echocardiographic findings, and hemodynamics of the study population (n = 487) according to the 2018 definition

	CpcPH (n = 65)	IpcPH (n = 140)	Precapillary PH (n = 16)	No PH (n = 266)	*p*‐value
Age (years)	79 ± 8	75 ± 10	80 ± 6	73 ± 10	<.001
Gender (male)	31 (48%)	85 (61%)	7 (44%)	156 (59%)	.21
Body mass index (kg/m^2^)	26.8 ± 4.4	29.3 ± 5.8	25.4 ± 5.4	27.5 ± 4.7	<.001
Body surface area (m^2^)	1.81 ± 0.18	1.94 ± 0.25	1.74 ± 0.27	1.88 ± 0.21	<.001
eGFR (ml/min/1.73m^2^)	61 ± 31	75 ± 31	70 ± 27	76 ± 27	.002
Hemoglobin (g/l)	130 ± 18	133 ± 17	128 ± 27	137 ± 16	.004
Diabetes	10 (15%)	41 (29%)	6 (38%)	41 (15%)	.002
Stroke	4 (6%)	10 (7%)	2 (13%)	13 (5%)	.55
Chronic obstructive lung disease	13 (20%)	10 (7%)	4 (25%)	31 (12%)	.02
FEV1 (% predicted)	77 ± 19	84 ± 18	74 ± 27	90 ± 20	<.001
Heart rhythm					<.001
Sinus rhythm	43 (66%)	116 (83%)	14 (87%)	248 (93%)	
Atrial fibrillation	18 (28%)	20 (14%)	2 (13%)	9 (3.5%)	
Pacemaker	4 (6%)	4 (3%)	0	9 (3.5%)	
Heart rate (bpm)	75 ± 15	72 ± 13	69 ± 7	67 ± 10	<.001
Medication					
Oral anticoagulation	26 (40%)	32 (23%)	2 (13%)	34 (13%)	<.001
Aspirin	32 (49%)	85 (61%)	12 (75%)	167 (63%)	.14
Loop diuretics	57 (88%)	84 (60%)	8 (50%)	91 (34%)	<.001
Betablocker	32 (49%)	75 (54%)	11 (69%)	111 (42%)	.04
ACEI/ARB	28 (43%)	88 (63%)	9 (56%)	145 (55%)	.07
Digoxin	14 (22%)	9 (6%)	2 (13%)	6 (2%)	<.001
Spironolactone	7 (11%)	9 (6%)	1 (6%)	7 (3%)	.04
B‐type natriuretic peptide (ng/l)	1010 (496–2050)	322 (182–523)	363 (175–638)	94 (51–188)	<.001
Symptoms					
Dyspnea NYHA class					<.001
I	5 (8%)	20 (14%)	2 (13%)	67 (25%)	
II	20 (31%)	70 (50%)	6 (37%)	146 (55%)	
III	30 (46%)	46 (33%)	7 (44%)	47 (18%)	
IV	10 (15%)	4 (3%)	1 (6%)	6 (2%)	
Mode of AVR					<.001
Surgical AVR	33 (51%)	88 (63%)	7 (44%)	218 (82%)	
Transcatheter AVR	32 (49%)	52 (37%)	9 (56%)	48 (18%)	
Echocardiography					
Left ventricular end‐diastolic diameter (mm)	48 ± 9	49 ± 8	47 ± 7	47 ± 7	.08
Left ventricular ejection fraction (%)	51 ± 14	54 ± 13	58 ± 9	61 ± 10	<.001
E/e'	24.0 ± 12.4	18.0 ± 8.3	23.5 ± 13.8	14.3.0 ± 6.1	<.001
Indexed left atrial area (cm^2^/m^2^)	14 ± 3	12 ± 3	12 ± 2	12 ± 3	<.001
TAPSE (mm)	18 ± 5	21 ± 5	19 ± 3	23 ± 5	<.001
Estimated sPAP (mmHg)	51 ± 14	40 ± 10	45 ± 13	34 ± 9	<.001
Mean aortic valve gradient (mmHg)	47 ± 19	47 ± 19	46 ± 17	47 ± 16	1.0
Aortic valve area (cm^2^)	0.70 ± 0.24	0.77 ± 0.21	0.73 ± 0.14	0.83 ± 0.24	<.001
Indexed aortic valve area (cm^2^/m^2^)	0.39 ± 0.13	0.40 ± 0.10	0.43 ± 0.12	0.44 ± 0.12	<.001
Aortic regurgitation (at least moderate)	12 (18%)	10 (7%)	1 (6%)	17 (6%)	.02
Mitral regurgitation					<.001
no	11 (17%)	44 (31%)	5 (31%)	171 (64%)	
mild	34 (52%)	82 (59%)	9 (56%)	82 (31%)	
moderate	16 (25%)	12 (9%)	2 (13%)	10 (4%)	
severe	4 (6%)	2 (1%)	0	3 (1%)	
Coronary artery disease					.85
No coronary artery disease	32 (49%)	70 (50%)	8 (50%)	145 (55%)	
1‐vessel disease	11 (17%)	22 (16%)	2 (12.5%)	50 (19%)	
2‐vessel disease	11 (17%)	20 (14%)	2 (12.5%)	33 (12%)	
3‐vessel disease	11 (17%)	28 (20%)	4 (25%)	38 (14%)	
Invasive haemodynamics					
Mean right atrial pressure (mmHg)	10 ± 5	8 ± 3	5 ± 2	5 ± 3	<.001
Right ventricular end‐diastolic pressure (mmHg)	12 ± 5	10 ± 4	8 ± 4	6 ± 3	<.001
sPAP (mmHg)	65 ± 14	44 ± 9	41 ± 8	31 ± 7	<.001
dPAP (mmHg)	26 ± 7	19 ± 5	15 ± 4	11 ± 4	<.001
mPAP (mmHg)	42 ± 9	30 ± 6	26 ± 4	19 ± 4	<.001
mPAWP (mmHg)	26 ± 7	22 ± 5	12 ± 3	11 ± 3	<.001
Transpulmonary gradient (mmHg)	16 ± 5	8 ± 3	14 ± 3	8 ± 3	<.001
Pulmonary vascular resistance (Wood units)	4.5 ± 1.5	1.7 ± 0.7	3.7 ± 0.8	1.7 ± 0.7	<.001
Diastolic pressure gradient (mmHg)	1 (−4–4)	‐3 (−5‐ ‐1)	5 (2–6)	0 (−1–2)	<.001
Pulmonary artery compliance (ml/mmHg)	1.5 ± 0.7	2.8 ± 1.3	2.3 ± 0.9	4.1 ± 1.9	<.001
Systolic aortic pressure (mmHg)	141 ± 29	150 ± 26	143 ± 23	144 ± 23	.04
Diastolic aortic pressure (mmHg)	67 ± 13	70 ± 11	68 ± 10	68 ± 11	.36
Mean aortic pressure (mmHg)	96 ± 16	101 ± 14	99 ± 13	97 ± 13	.03
Systemic vascular resistance (Wood units)	23.5 ± 5.8	20.3 ± 5.3	25.0 ± 8.1	19.3 ± 4.2	<.001
Arterial oxygen saturation (%)	94 (92–96)	95 (93–97)	94 (92–95)	96 (94–97)	<.001
Mixed venous oxygen saturation (%)	62 (56–65)	68 (62–71)	66 (59–69)	70 (67–73)	<.001
Cardiac output (L/min)	3.8 ± 0.7	4.8 ± 1.1	3.9 ± 0.9	4.9 ± 0.9	<.001
Cardiac index (L/min/m^2^)	2.1 ± 0.4	2.5 ± 0.5	2.3 ± 0.6	2.6 ± 0.4	<.001
Stroke volume index (ml/m^2^)	29 ± 9	36 ± 10	34 ± 9	40 ± 8	<.001

*Note:* Data are given as numbers and percentages, mean ± *SD*, or median (interquartile range).

Abbreviations: ACEI/ARB, angiotensin converting enzyme inhibitor/angiotensin receptor blocker, AVR, aortic valve replacement; E/e', ratio of peak early mitral inflow velocity to peak early mitral annular velocity, eGFR, estimated glomerular filtration rate; FEV1, forced expiratory volume within the first second; mPAP, mean pulmonary artery pressure; mPAWP, mean pulmonary artery wedge pressure; NYHA, New York Heart Association; sPAP, systolic pulmonary artery pressure; TAPSE, tricuspid annular plane systolic excursion.

### 3.4 Reclassification

**FIGURE 1:**
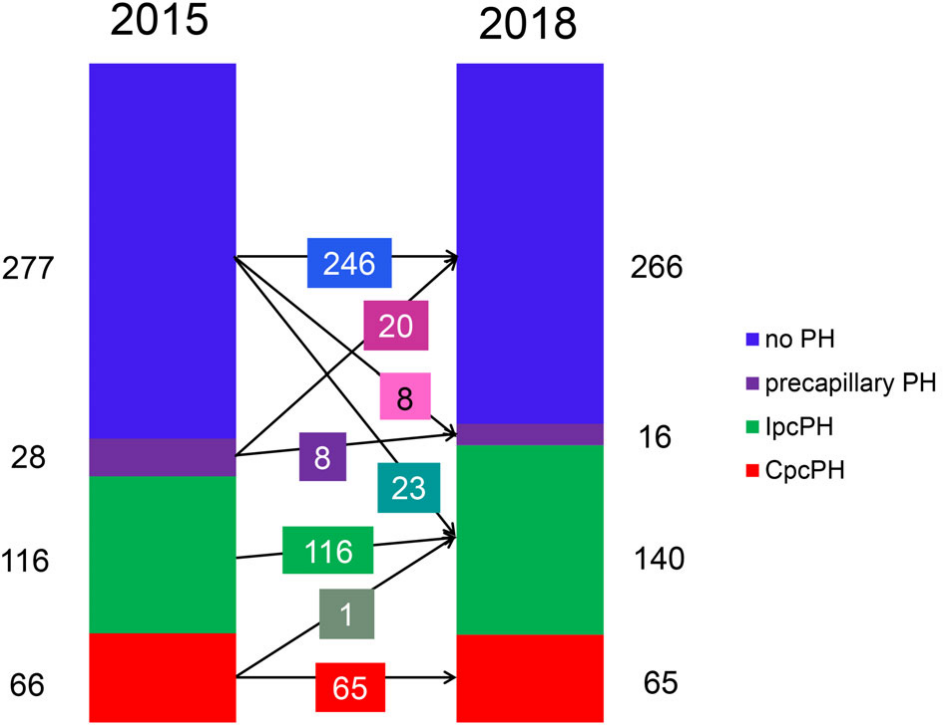
Bar graph showing the proportion of patients (n = 487) with combined pre and postcapillary pulmonary hypertension (CpcPH), isolated postcapillary PH (IpcPH), precapillary PH, and without PH according to the 2015 and 2018 definitions and the reclassification steps

Overall, 52 (11%) patients out of 487 were reclassified after applying the 2018 definition instead of 2015 definition, that is, 23 patients from no PH to IpcPH, eight from no PH to precapillary PH, 20 from precapillary PH to no PH, and one patient from CpcPH to IpcPH (Figure 1). Concerning the 20 patients that were reclassified from precapillary PH to no PH, their mPAP was 26 ± 1 mmHg, mPAWP 14 ± 1 mmHg, transpulmonary gradient 12 ± 2 mmHg, and PVR 2.4 ± 0.4 WU (Figure S1). Indexed aortic valve area, left ventricular ejection fraction, and key hemodynamic parameters in patients with different reclassification scenarios and patients who were not reclassified are shown in the Figure S1.

### 3.5 Prognostic impact of the 2015 versus 2018 definition

**FIGURE 2:**
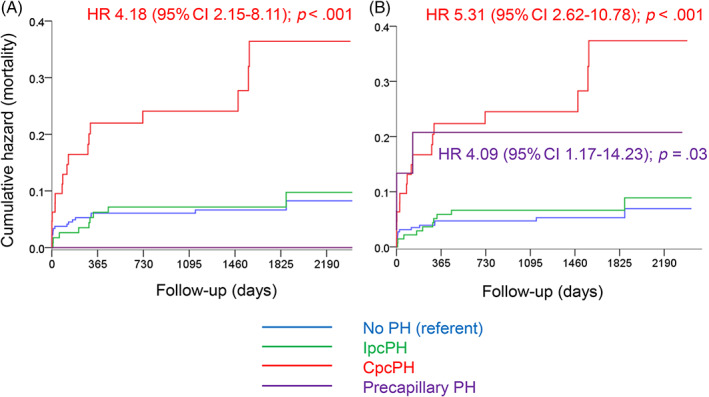
Kaplan Meier plots showing cumulative events (mortality) for patients with combined pre and postcapillary pulmonary hypertension (CpcPH), isolated postcapillary PH (IpcPH), precapillary PH, and no PH according to the 2015 (Panel A) and 2018 (Panel B) definitions. HR:, hazard ratio; 95% CI, 95% confidence interval

The 30‐day mortality was 4.1% (20/487 patients). After a median post‐AVR follow‐up of 44 (31–62) months, 44 deaths had occurred. When applying the 2015 definition, CpcPH patients experienced a four‐fold higher mortality compared to those without PH (referent), whereas mortality did not significantly differ among patients with IpcPH, precapillary PH, and no PH at all (Figure 2(A)). In contrast, when applying the 2018 definition, both CpcPH and precapillary PH patients displayed a more than four‐fold higher mortality compared to those without PH (Figure 2(B)). According to both definitions, any PH was associated with increased long‐term mortality after AVR (Figure S2). Overall, reclassified patients had a similar mortality compared to nonreclassified patients (Figure S3).

### 3.6 Prognostic impact of different hemodynamic cut‐offs

**FIGURE 3:**
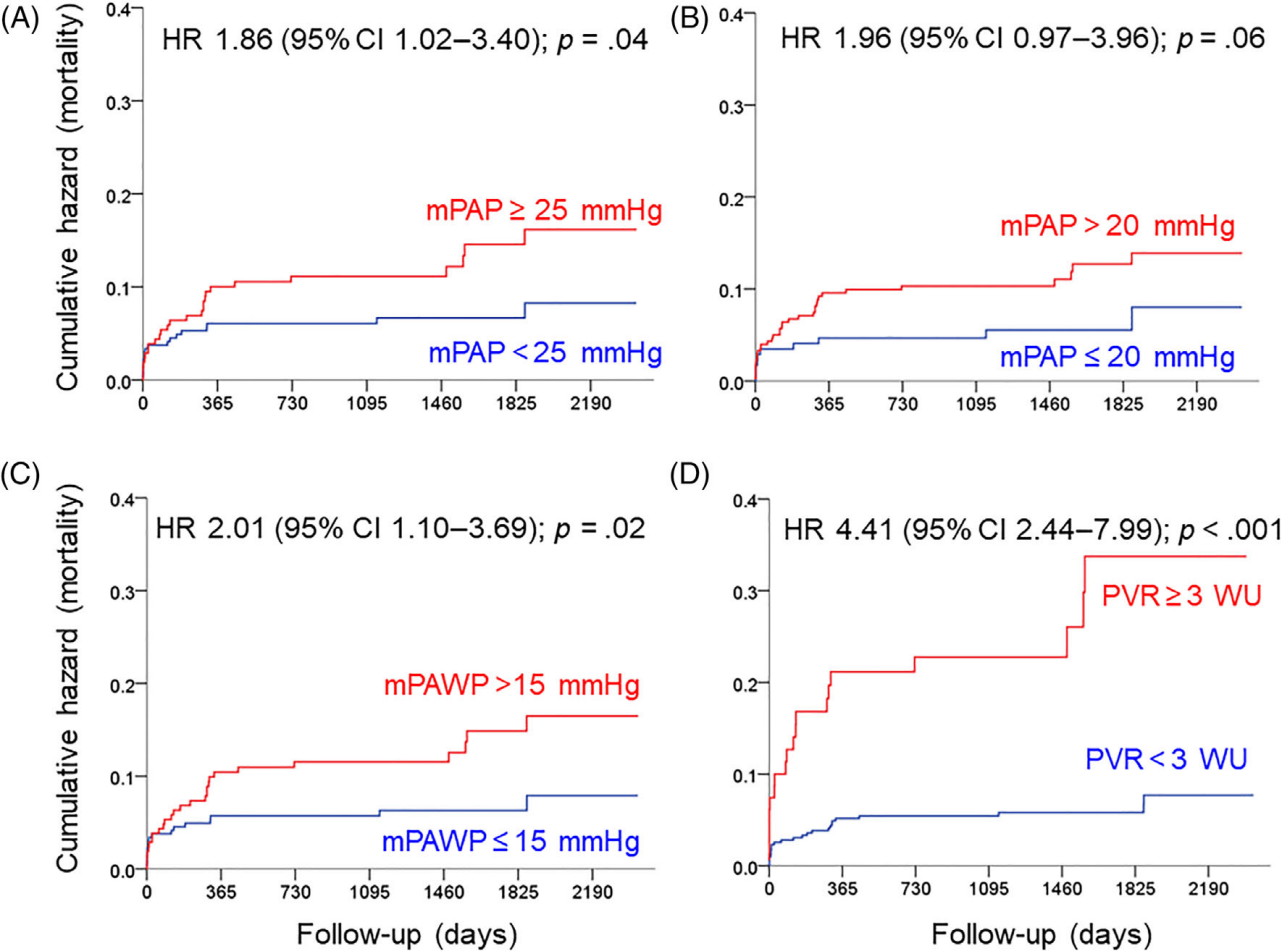
Kaplan Meier plots showing cumulative events (mortality) according to cut‐offs of single hemodynamic parameters. mPAP, mean pulmonary artery pressure; mPAWP, mean pulmonary artery wedge pressure; PVR, pulmonary vascular resistance; HR, hazard ratio, 95% CI, 95% confidence interval

**FIGURE 4:**
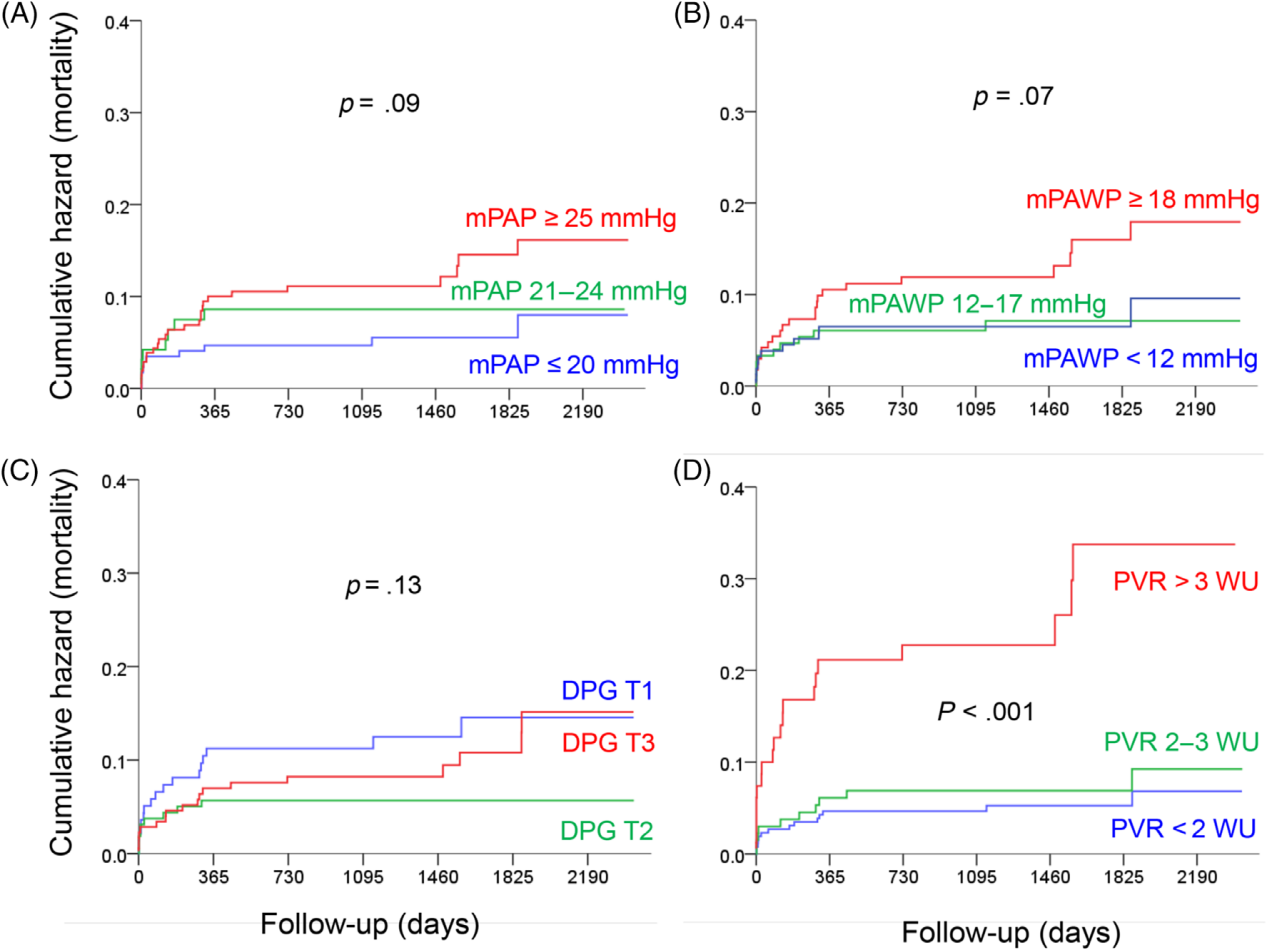
Kaplan Meier plots showing cumulative events (mortality) according to single hemodynamic parameters [three categories; tertiles for mean pulmonary artery wedge pressure (mPAWP) and diastolic pressure gradient (DPG), cut‐offs of interest for mean pulmonary artery pressure (mPAP), and pulmonary vascular resistance (PVR)]. HR: hazard ratio, 95%CI: 95% confidence interval

The prognostic impact of the single hemodynamic parameters contributing to the PH definitions is shown in Figure 3. Both mPAP cut‐offs [≥25 vs. <25 mmHg (panel A) and > 20 vs. ≤20 mmHg (panel B)] and the currently used mPAWP cut‐off (>15 vs. ≤15mmH; panel C) were associated with a hazard ratio for mortality of ~2.0, whereas the hazard ratio for PVR ≥3 versus <3 WU was 4.4 (Panel D). Patients with mPAP 21‐24 mmHg (i.e., patients falling into the new mPAP range that represents part of the 2018 PH definition) displayed similar mortality as those with mPAP ≤20 mmHg (Figure 4(A)). In Figure 4, panels B–D, mortality according to three categories for mPAWP, DGP, and PVR is shown, while evaluating the potential utility of cut‐offs other than the established ones. The risk of death was particularly high in patients with the highest mPAWP tertile (≥18 mmHg; B); this risk was not related to DPG (C), but markedly increased in patients with PVR >3WU, yet not in those with PVR 2‐3WU (D).

## 4 DISCUSSION

This first study analyzing the impact of the 2018 PH definition^3^ in a large cohort of severe AS patients revealed the following: (1) 11% of patients were differently classified according to the 2018 versus 2015 definition; (2) classification changes mainly resulted in new IpcPH cases, as well as changes from precapillary PH to no PH cases and vice versa; (3) in contrast to the 2015 definition, patients with precapillary PH according to the 2018 definition exhibited increased mortality post‐AVR; (4) PVR ≥3WU was found to be the key driver of an increased post‐AVR mortality.

Very little data have been published so far to illustrate the new PH definition's potential implications.^15^, ^16^, ^17^ Among 268 patients with systemic sclerosis, 89 patients exhibited precapillary PH, 29 IpcPH, 19 CpcPH, whereas 131 were free of any PH according to the 2015 definition.^15^ Based on the 2018 definition, seven additional patients had PH (reclassification rate of 3%). Of these, three displayed IpcPH and four precapillary PH.^15^ In a cohort of 630 patients with mPAWP ≤15 mmHg, Kovacs et al.^16^ recently found that 281 patients neither met the 2015 nor 2018 criteria for precapillary PH, 30 patients only met the 2015 criteria, 30 patients only met the 2018 criteria, and 289 patients met both criteria. Patients only fulfilling the 2018 criteria had mild forms of group 1, 3, or 4 PH, and had higher mortality (median follow‐up of 8 years) than patients meeting neither the 2015 nor the 2018 criteria, whereas patients only fulfilling the 2015 criteria had similar mortality as those without PH.^16^

The setting of our study differed from these studies,^15^, ^16^ as we examined a population with a high postcapillary PH prevalence. However, while all AS patients have a predisposition for postcapillary PH, additional pathologies can still be present.^18^, ^25^ In our 487 AS patients, we found a reclassification rate exceeding 10%. On the one hand, the reclassified group comprised 23 patients reclassified from no PH to IpcPH. These patients showed an mPAWP >15 mmHg, yet very low PVR, and therefore an mPAP between 21 and 24 mmHg. On the other hand, eight patients were reclassified from having no PH at all into precapillary PH group. These patients exhibited a PVR ≥3 WU, yet along with a low mPAWP and mPAP between 21 and 24 mmHg, thus failing to meet the 2015 criteria^1^ for precapillary PH. Most of the other patients (n = 20) were reclassified from precapillary PH into no PH: These patients had an mPAP of 25 mmHg or slightly more, mPAWP of 15 mmHg or slightly less, relatively high cardiac output, and thus PVR <3 WU. According to the 2015 definition that is devoid of any PVR criterion for defining precapillary PH,^1^ these patients were labeled as precapillary PH due to mPAWP ≤15 mmHg. In contrast, these patients did not meet the 2018 definition of precapillary PH,^3^ because the PVR ≥3 WU criterion was not fulfilled. These cases may have been “occult” IpcPH patients with borderline mPAWP on account of fasting or diuretic therapy.^26^ This remains speculative however given that systematic and standardized volume challenge testing was not performed. This group of patients with mPAP >20 mmHg, mPAWP ≤15 mmHg, and PVR <3 WU is not explicitly addressed by the 2018 definition^3^ although it is stated that a mPAP of 20 mmHg should be considered the upper limit of normal value. Given that these patients neither meet precapillary nor postcapillary criteria of the 2018 definition we assumed that they do not have PH. The next guidelines will probably address this issue. Our interpretation of a “no PH” label may be controversial but is supported by the favorable hemodynamic profile and prognosis of these patients. In the only other available study focusing on group 2 PH, 58 patients from a cohort of 726 patients undergoing cardiac catheterization were found to have post‐capillary PH according to the 2015 definition, whereas 59 (+1 patient) had post‐capillary PH according to the 2018 definition. This study found an increase in the proportion of CpcPH patients when applying the 2018 definition (from 34.4 to 64.4%). However, in this study CpcPH according to the 2015 definition was defined by the strict PVR >3 WU and DPG ≥7 mmHg criterion^17^ which had previously been proposed^27^, ^28^ (but not supported any more 2 years later^29^) with the intention to avoid unclassifiable patients and to select “true” CpcPH patients. However, this study included considerably less post‐capillary PH patients than the present one and did not report outcome data.^17^ As outlined above based on more recent prognostic data^24^ we applied the PVR >3 WU and/or DPG ≥7 mmHg criterion for the 2015 definition of CpcPH, and the poor prognosis of CpcPH patients selected by this criterion (Figure 3(A)) seems to support our practice.

One of the strengths of the current study is that this is the only available study in a group 2 PH setting examining hemodynamic data in relation to long‐term prognostic information. According to both the 2015^1^ and 2018^3^ definitions, mortality of IpcPH patients did not differ from that of patients without any PH. Importantly, patients “without PH” (comparator group) were not to be seen as normal subjects, as they also exhibited a left ventricle that was exposed to chronic pressure overload. In IpcPH patients, the AVR‐induced afterload reduction was likely able to induce favorable left ventricular and left atrial remodeling with reduction of mPAWP over time, which may explain the favorable post‐AVR outcome. In contrast, patients with established pulmonary vascular involvement (PVR ≥3 WU) displayed a four‐fold higher risk of death (patients with CpcPH according to the 2015^1^ and 2018^3^ definitions or precapillary PH according to the 2018 definition^3^). Thus, for this subset of patients that were reclassified from normal to precapillary PH and from precapillary PH to normal, the application of the new diagnosis is clinically crucial. The strong prognostic impact of PVR is in line with the literature.^14^ The present data confirm this in the context of the application of the 2015 versus the 2018 definitions in patients with AS.

### 4.1 Limitations

First, the number of patients in the different PH groups was relatively small and too low to come to a definite conclusion regarding the new definition's prognostic impact. Although we found similar mortality in patients with PVR <2 WU and those with PVR 2–3 WU, it remains possible that the risk of death may be rising at PVR values less than 3 as shown for other populations.^14^ Nevertheless, this is one of the first larger‐scale studies designed to assess the possible impact of the new PH definition including its prognostic implications and the first one in AS patients as a typical example of an important left heart disease. Second, to assess cardiac output, we have employed the indirect Fick method, which may be subject to error, as oxygen consumption is often inaccurately estimated.^30^ This has implication for the calculation of PVR, that is, an underestimation of PVR if cardiac output it overestimated and vice versa. It must, however, be noted that this technique is routinely used in clinical practice. Third, we acknowledge that mPAWP results may vary depending on the methodology, for example, on the measurement during the respiratory cycle (end‐expiratory versus averaging over several cycles).^31^ Experts agree that averaging mPAWP over three respiratory cycles is also acceptable as opposed to end‐expiratory measurements.^32^ Finally, our assumption that the patients with mPAP >20 mmHg, mPAWP ≤15 mmHg, and PVR <3 WU, which is not explicitly addressed in the 2018 definition paper, can be labeled as “no PH” may be subject to discussion as this is not explicitly stated either. We hope that this will be clarified in future recommendations and guidelines.

### 4.2 Conclusions

In severe AS, the new PH definition diagnoses a higher number of patients as having IpcPH, even though their prognosis is similar to that observed in patients without any PH. Patients with true precapillary PH are more accurately identified by means of the 2018 proposal that includes a pulmonary vascular disease criterion defined as PVR ≥3WU, which is a strong predictor of poor prognosis.

## Supporting information

**Figure S1** Indexed aortic valve area (AVAi), left ventricular ejection fraction (LVEF), and key hemodynamic parameters in patients with different reclassification scenarios and patients who were not reclassified. Error bars represent means and standard deviations. CO, cardiac output; CpcPH, combined pre and postcapillary pulmonary hypertension; IpcPH, isolated postcapillary pulmonary hypertension; mPAP, mean pulmonary artery pressure; mPAWP, mean pulmonary artery wedge pressure; PH, pulmonary hypertension; PVR, pulmonary vascular resistance. The scale is: mm^2^/m^2^, %, mmHg, Wood units*10, and l/min*10.

**Figure S2** Kaplan Meier plots showing cumulative events (mortality) in patients with any pulmonary hypertension (PH) according to the 2015 (panel A) and the 2018 (panel B) definition. HR, hazard ratio; 95% CI, 95% confidence interval.

**Figure S3** Kaplan Meier plots showing cumulative events (mortality) in patients who were reclassified and who were no. HR, hazard ratio; 95% CI, 95% confidence interval.

**Table S1** Clinical characteristics, echocardiographic findings, and hemodynamics of the study population (n = 487) according to the 2015 definition.

## Data Availability

The data that support the findings of this study are available from the corresponding author (MTM) upon reasonable request.
