# Supplementary figures and images for: Impact of the new pulmonary hypertension definition on long‐term mortality in patients with severe aortic stenosis undergoing valve replacement
Source: Clin Cardiol. 2021 Jul 4;44(9):1276–85. doi: 10.1002/clc.23685 (PMC8428068; doi:10.1002/clc.23685)

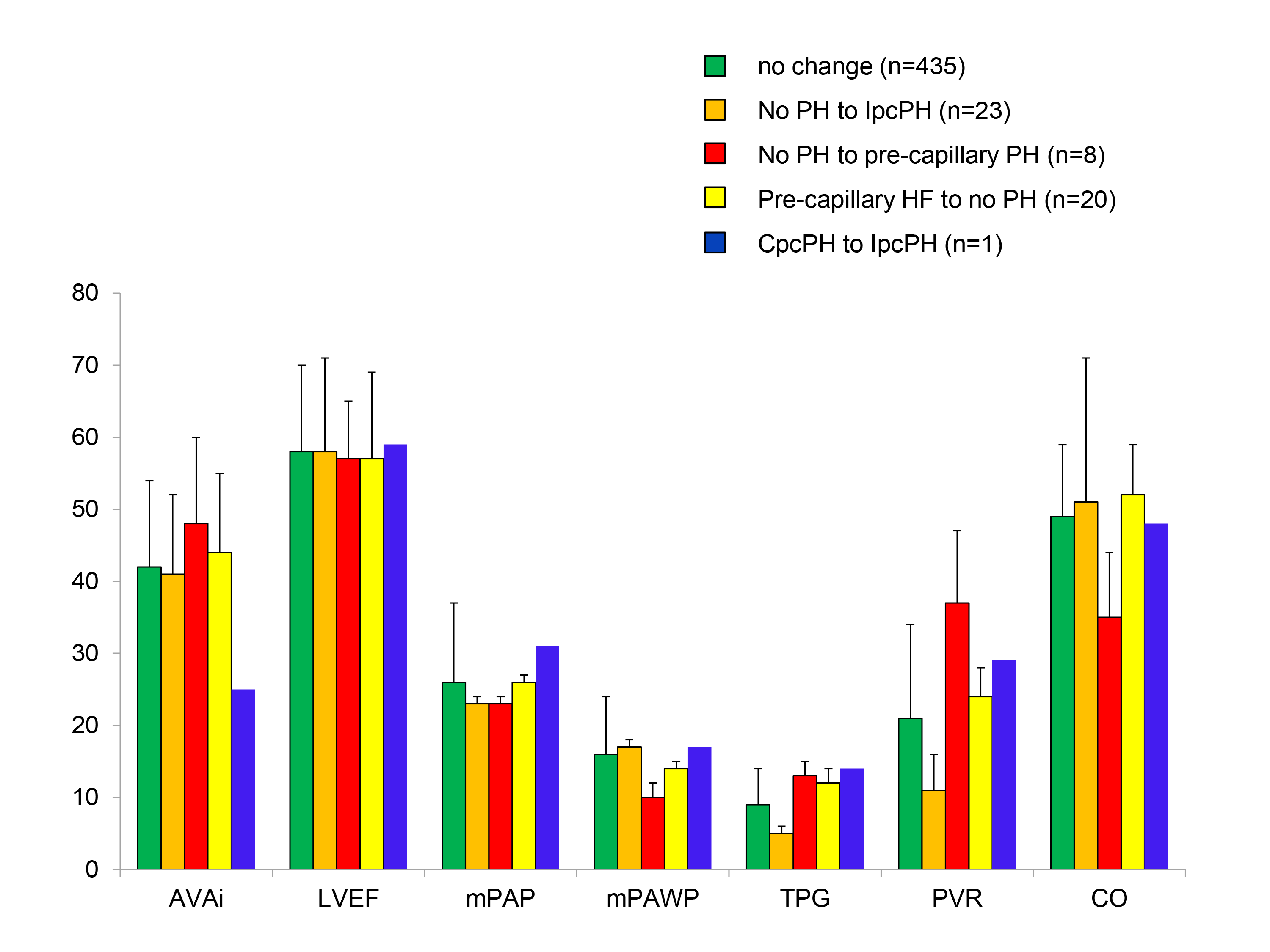

Supplement: Supplementary file 1 — Figure S1 Indexed aortic valve area (AVAi), left ventricular ejection fraction (LVEF), and key hemodynamic parameters in patients with different reclassification scenarios and patients who were not reclassified. Error bars represent means and standard deviations. CO, cardiac output; CpcPH, combined pre and postcapillary pulmonary hypertension; IpcPH, isolated postcapillary pulmonary hypertension; mPAP, mean pulmonary artery pressure; mPAWP, mean pulmonary artery wedge pressure; PH, pulmonary hypertension; PVR, pulmonary vascular resistance. The scale is: mm2/m2, %, mmHg, Wood units*10, and l/min*10. [file CLC-44-1276-s003.tif]

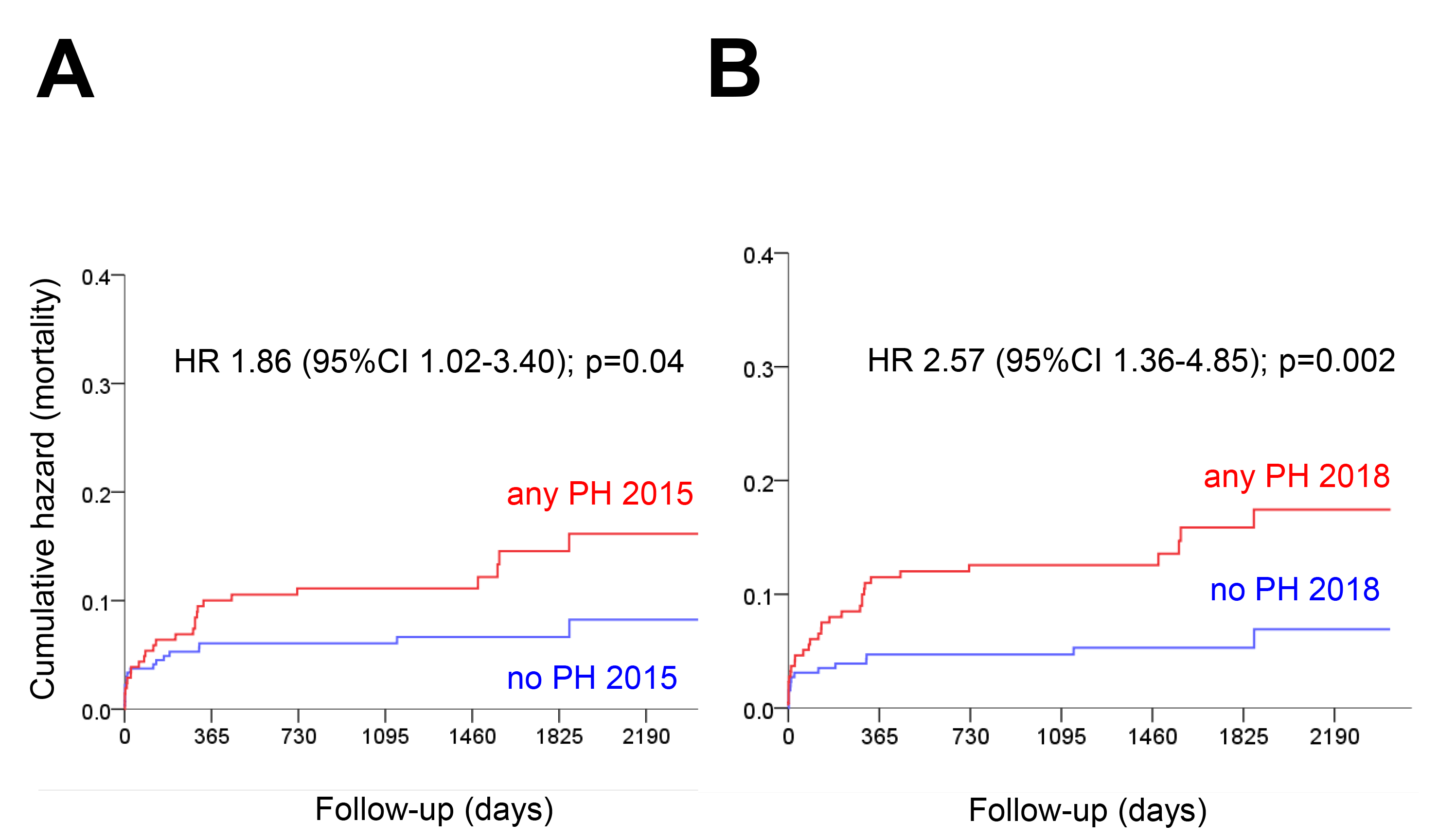

Supplement: Supplementary file 2 — Figure S2 Kaplan Meier plots showing cumulative events (mortality) in patients with any pulmonary hypertension (PH) according to the 2015 (panel A) and the 2018 (panel B) definition. HR, hazard ratio; 95% CI, 95% confidence interval. [file CLC-44-1276-s001.tif]

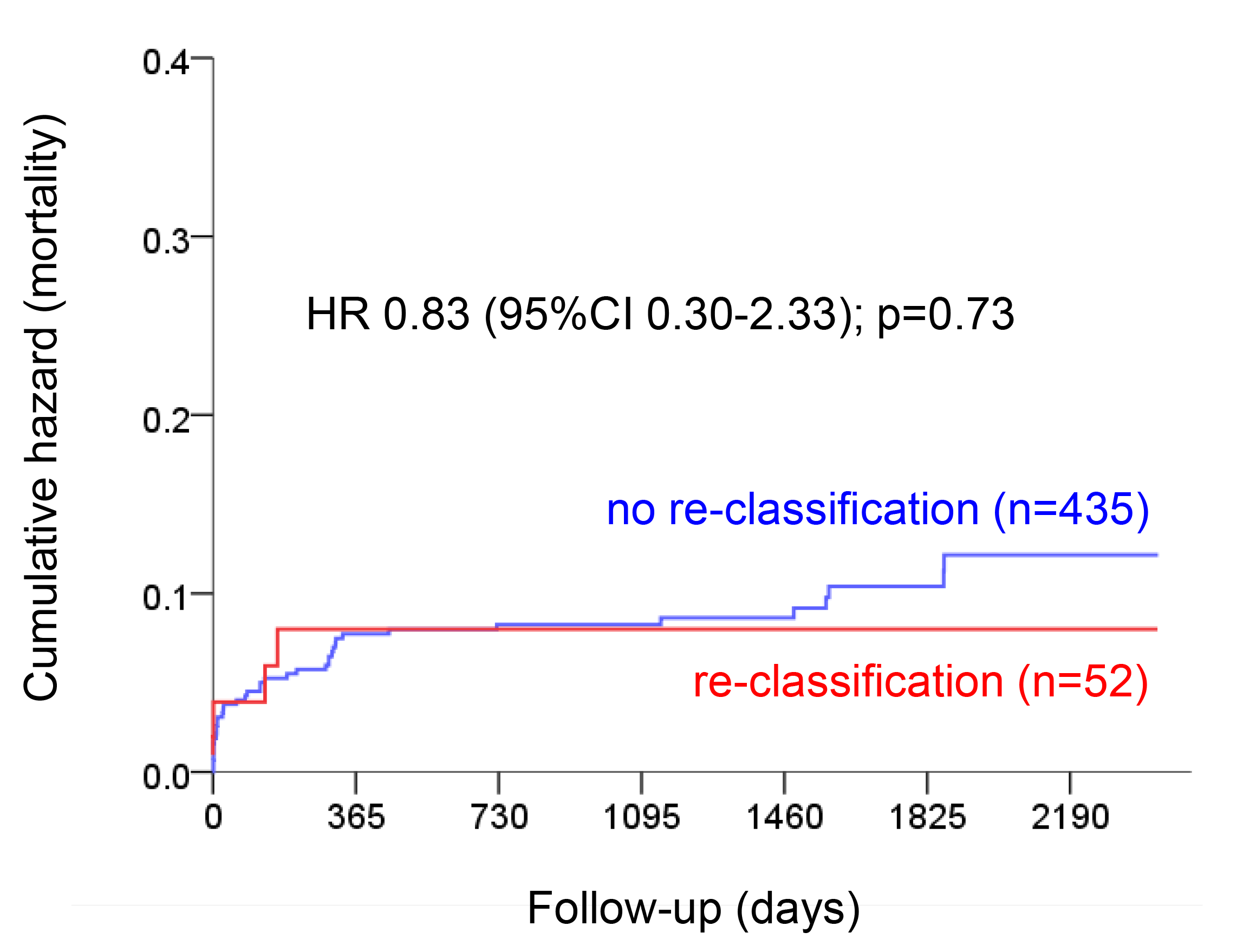

Supplement: Supplementary file 3 — Figure S3 Kaplan Meier plots showing cumulative events (mortality) in patients who were reclassified and who were no. HR, hazard ratio; 95% CI, 95% confidence interval. [file CLC-44-1276-s002.tif]
