# Supplementary material for: Impact of the new pulmonary hypertension definition on long‐term mortality in patients with severe aortic stenosis undergoing valve replacement
Source: Clin Cardiol. 2021 Jul 4;44(9):1276–85. doi: 10.1002/clc.23685 (PMC8428068; doi:10.1002/clc.23685)
Supplement: Supplementary file 4 — Table S1 Clinical characteristics, echocardiographic findings, and hemodynamics of the study population (n = 487) according to the 2015 definition. [file CLC-44-1276-s004.docx]

**Supplementary Material**

**Supplementary Table 1.** Clinical characteristics, echocardiographic findings, and hemodynamics of the study population (n=487) according to the 2015 definition

|  | **CpcPH (n=66)** | **IpcPH**  **(n=116)** | **Pre-capillary PH (n=28)** | **No PH**  **(n=277)** | **P value** |
| --- | --- | --- | --- | --- | --- |
| Age (years) | 78±9 | 76±9 | 75±9 | 73±10 | <0.001 |
| Gender (male) | 32 (48%) | 67 (58%) | 17 (61%) | 163 (59%) | 0.47 |
| Body mass index (kg/m^2^) | 26.8±4.4 | 29.5±5.8 | 29.0±5.7 | 27.3±4.7 | <0.001 |
| Body surface area (m^2^) | 1.82±0.18 | 1.94±0.24 | 1.90±0.24 | 1.87±0.22 | 0.002 |
| eGFR (ml/min/1.73m^2^) | 62±31 | 74±30 | 76±30 | 76±27 | 0.005 |
| Hemoglobin (g/l) | 131±18 | 132±18 | 132±25 | 137±16 | 0.02 |
| Diabetes | 10 (15%) | 34 (29%) | 6 (21%) | 48 (17%) | 0.04 |
| Stroke | 4 (6%) | 8 (7%) | 2 (7%) | 15 (5%) | 0.94 |
| Chronic obstructive lung disease | 13 (20%) | 9 (8%) | 8 (29%) | 28 (10%) | 0.003 |
| FEV1 (% predicted) | 77±19 | 83±19 | 76±23 | 91±20 | <0.001 |
| **Heart rhythm** |  |  |  |  | <0.001 |
| Sinus rhythm | 44 (67%) | 95 (82%) | 27 (96%) | 255 (92%) |  |
| Atrial fibrillation | 18 (27%) | 17 (15%) | 1 (4%) | 13 (5%) |  |
| pacemaker | 4 (6%) | 4 (3%) | 0 | 9 (3%) |  |
| Heart rate (bpm) | 75±15 | 73±13 | 70±8 | 67±11 | <0.001 |
| **Medication** |  |  |  |  |  |
| Oral anticoagulation | 26 (39%) | 29 (25%) | 2 (7%) | 37 (13%) | <0.001 |
| Aspirin | 32 (48%) | 68 (59%) | 21 (75%) | 175 (63%) | 0.06 |
| Loop diuretics | 58 (88%) | 72 (62%) | 13 (46%) | 97 (35%) | <0.001 |
| Betablocker | 32 (48%) | 63 (54%) | 17 (61%) | 117 (42%) | 0.07 |
| ACEI/ARB | 28 (42%) | 73 (63%) | 19 (68%) | 150 (54%) | 0.03 |
| Digoxin | 14 (21%) | 9 (8%) | 2 (7%) | 6 (2%) | <0.001 |
| Spironolactone | 7 (11%) | 8 (7%) | 0 | 9 (3%) | 0.04 |
| B-type natriuretic peptide (ngL) | 1010 (496-2050) | 348 (180-556) | 158 (104-374) | 105 (55-208) | <0.001 |
| **Symptoms** |  |  |  |  |  |
| Dyspnea NYHA class |  |  |  |  | <0.001 |
| I | 5 (8%) | 15 (13%) | 1 (3.5%) | 73 (26%) |  |
| II | 21 (32%) | 56 (48%) | 14 (50%) | 151 (55%) |  |
| III | 30 (45%) | 42 (36%) | 12 (43%) | 46 (17%) |  |
| IV | 10 (15%) | 3 (3%) | 1 (3.5%) | 7 (2%) |  |
| **Mode of AVR** |  |  |  |  | <0.001 |
| Surgical AVR | 34 (52%) | 69 (59%) | 17 (61%) | 226 (82%) |  |
| Transcatheter AVR | 32 (48%) | 47 (41%) | 11 (39%) | 51 (18%) |  |
| **Echocardiography** |  |  |  |  |  |
| Left ventricular end-diastolic diameter (mm) | 48±9 | 48±8 | 48±9 | 47±7 | 0.42 |
| Left ventricular ejection fraction (%) | 51±14 | 54±13 | 58±11 | 61±10 | <0.001 |
| E/e’ | 23.6±12.3 | 18.0±8.0 | 16.6±7.7 | 14.9.0±7.1 | <0.001 |
| Indexed left atrial area (cm^2^/m^2^) | 17±5 | 14±3 | 13±4 | 12±3 | <0.001 |
| TAPSE (mm) | 18±5 | 21±5 | 19±4 | 23±5 | <0.001 |
| Estimated sPAP (mmHg) | 51±14 | 40±11 | 42±14 | 34±9 | <0.001 |
| Mean aortic valve gradient (mmHg) | 48±19 | 46±17 | 51±15 | 47±17 | 0.61 |
| Aortic valve area (cm^2^) | 0.69±0.24 | 0.77±0.20 | 0.80±0.19 | 0.82±0.25 | 0.001 |
| Indexed aortic valve area (cm^2^/m^2^) | 0.38±0.13 | 0.40±0.10 | 0.43±0.11 | 0.44±0.12 | 0.001 |
| Aortic regurgitation (at least moderate) | 12 (18%) | 9 (8%) | 1 (4%) | 18 (6%) | 0.02 |
| Mitral regurgitation |  |  |  |  | <0.001 |
| no | 11 (17%) | 35 (30%) | 17 (61%) | 168 (61%) |  |
| mild | 35 (53%) | 68 (59%) | 7 (25%) | 97 (35%) |  |
| moderate | 16 (24%) | 12 (10%) | 4 (14%) | 8 (3%) |  |
| severe | 4 (6%) | 1 (1%) | 0 | 4 (1%) |  |
| **Coronary artery disease** |  |  |  |  | 0.48 |
| No coronary artery disease | 33 (50%) | 57 (49%) | 11 (39%) | 154 (56%) |  |
| 1-vessel disease | 11 (16.6%) | 17 (15%) | 6 (22%) | 51 (18%) |  |
| 2-vessel disease | 11 (16.6%) | 18 (16%) | 4 (14%) | 33 (12%) |  |
| 3-vessel disease | 11 (16.6%) | 24 (21%) | 7 (25%) | 39 (14%) |  |
| **Invasive hemodynamics** |  |  |  |  |  |
| Mean right atrial pressure (mmHg) | 10±5 | 8±3 | 6±3 | 5±3 | <0.001 |
| Right ventricular end-diastolic pressure (mmHg) | 12±5 | 10±4 | 8±4 | 6±3 | <0.001 |
| sPAP (mmHg) | 64±14 | 47±9 | 43±12 | 30±6 | <0.001 |
| dPAP (mmHg) | 26±7 | 20±5 | 16±3 | 11±3 | <0.001 |
| mPAP (mmHg) | 42±9 | 32±5 | 27±3 | 19±4 | <0.001 |
| mPAWP (mmHg) | 26±7 | 23±5 | 13±2 | 11±4 | <0.001 |
| Transpulmonary gradient (mmHg) | 16±5 | 9±3 | 13±3 | 8±3 | <0.001 |
| Pulmonary vascular resistance (Wood units) | 4.5±1.5 | 1.8±0.7 | 2.8±0.8 | 1.6±0.7 | <0.001 |
| Diastolic pressure gradient (mmHg) | 1 (-3-4) | -3 (-6- -1) | 2 (0-5) | 0 (-2-1) | <0.001 |
| Pulmonary artery compliance (ml/mmHg) | 1.6±0.7 | 2.6±1.0 | 3.0±1.2 | 4.1±1.9 | <0.001 |
| Left ventricular end-diastolic pressure (mmHg) (n=335) | 25±7 | 26±8 | 21±7 | 19±7 | <0.001 |
| Systolic aortic pressure (mmHg) | 141±29 | 152±26 | 145±24 | 144±23 | 0.008 |
| Diastolic aortic pressure (mmHg) | 67±13 | 70±11 | 70±10 | 68±11 | 0.29 |
| Mean aortic pressure (mmHg) | 96±16 | 102±15 | 100±13 | 97±13 | 0.007 |
| Systemic vascular resistance (Wood units) | 23.5±5.8 | 20.5±5.3 | 19.4±3.6 | 19.6±4.9 | <0.001 |
| Arterial oxygen saturation (%) | 94 (92-96) | 95 (93-97) | 94 (92-95) | 96 (94-97) | 0.001 |
| Mixed venous oxygen saturation (%) | 62 (56-65) | 67 (62-72) | 68 (59-71) | 70 (67-73) | <0.001 |
| Cardiac output (l/min) | 3.8±0.7 | 4.7±0.9 | 4.9±0.8 | 4.9±1.1 | <0.001 |
| Cardiac index (l/min/m^2^) | 2.1±0.4 | 2.4±0.4 | 2.6±0.5 | 2.6±0.5 | <0.001 |
| Stroke volume index (ml/m^2^) | 29±9 | 35±9 | 38±7 | 40±9 | <0.001 |

Data are given as numbers and percentages, mean±standard deviation, or median (interquartile range).

ACEI/ARB = angiotensin converting enzyme inhibitor/angiotensin receptor blocker, AVR = aortic valve replacement, E/e’ = ratio of peak early mitral inflow velocity to peak early mitral annular velocity, eGFR = estimated glomerular filtration rate: FEV1 = forced expiratory volume within the first second, mPAP = mean pulmonary artery pressure; mPAWP = mean pulmonary artery wedge pressure, NYHA = New York Heart Association, sPAP = systolic pulmonary artery pressure, TAPSE = tricuspid annular plane systolic excursion.

**Legend to Supplementary Figures**

**Supplementary Figure 1.** Indexed aortic valve area (AVAi), left ventricular ejection fraction (LVEF), and key hemodynamic parameters in patients with different re-classification scenarios and patients who were not re-classified.

Error bars represent means and standard deviations.

CO: cardiac output, CpcPH: combined pre- and post-capillary pulmonary hypertension, IpcPH: isolated post-capillary pulmonary hypertension, mPAP: mean pulmonary artery pressure, mPAWP: mean pulmonary artery wedge pressure, PH: pulmonary hypertension, PVR: pulmonary vascular resistance.

The scale is: mm^2^/m^2^, %, mmHg, Wood units*10, and l/min*10.

**Supplementary Figure 2.** Kaplan Meier plots showing cumulative events (mortality) in patients with any pulmonary hypertension (PH) according to the 2015 (panel A) and the 2018 (panel B) definition.

HR: hazard ratio, 95%CI: 95% confidence interval

**Supplementary Figure 3.** Kaplan Meier plots showing cumulative events (mortality) in patients who were re-classified and who were no.

HR: hazard ratio, 95%CI: 95% confidence interval
